# Supplementary figures and images for: Ligand-induced shifts in conformational ensembles that describe transcriptional activation
Source: eLife. 2022 Oct 12;11:e80140. doi: 10.7554/eLife.80140 (PMC9555869; doi:10.7554/eLife.80140)

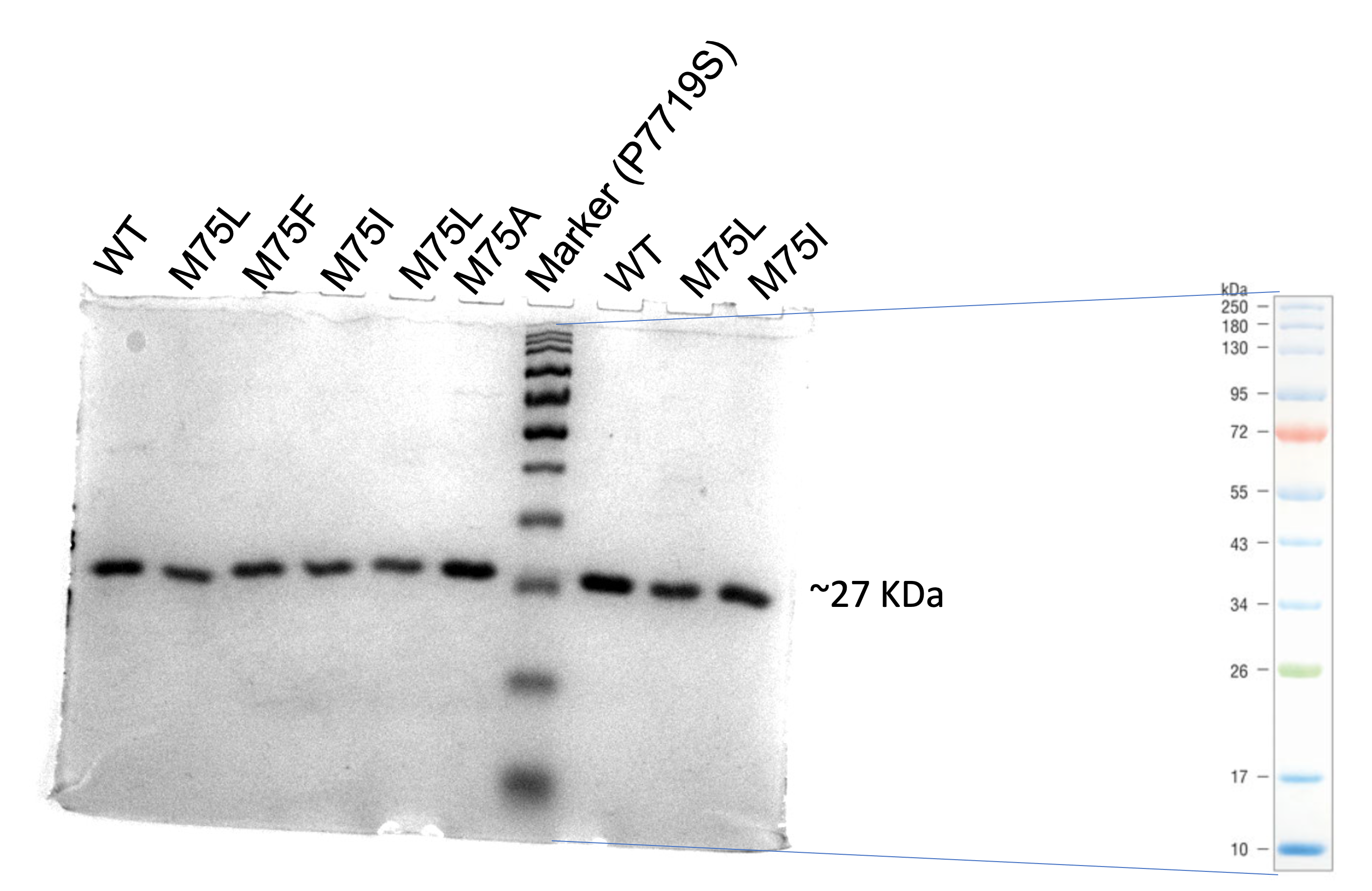

Supplement: Figure 1—figure supplement 1—source data 1. [file elife-80140-fig1-figsupp1-data1.zip › SDSgel_labelled.png]

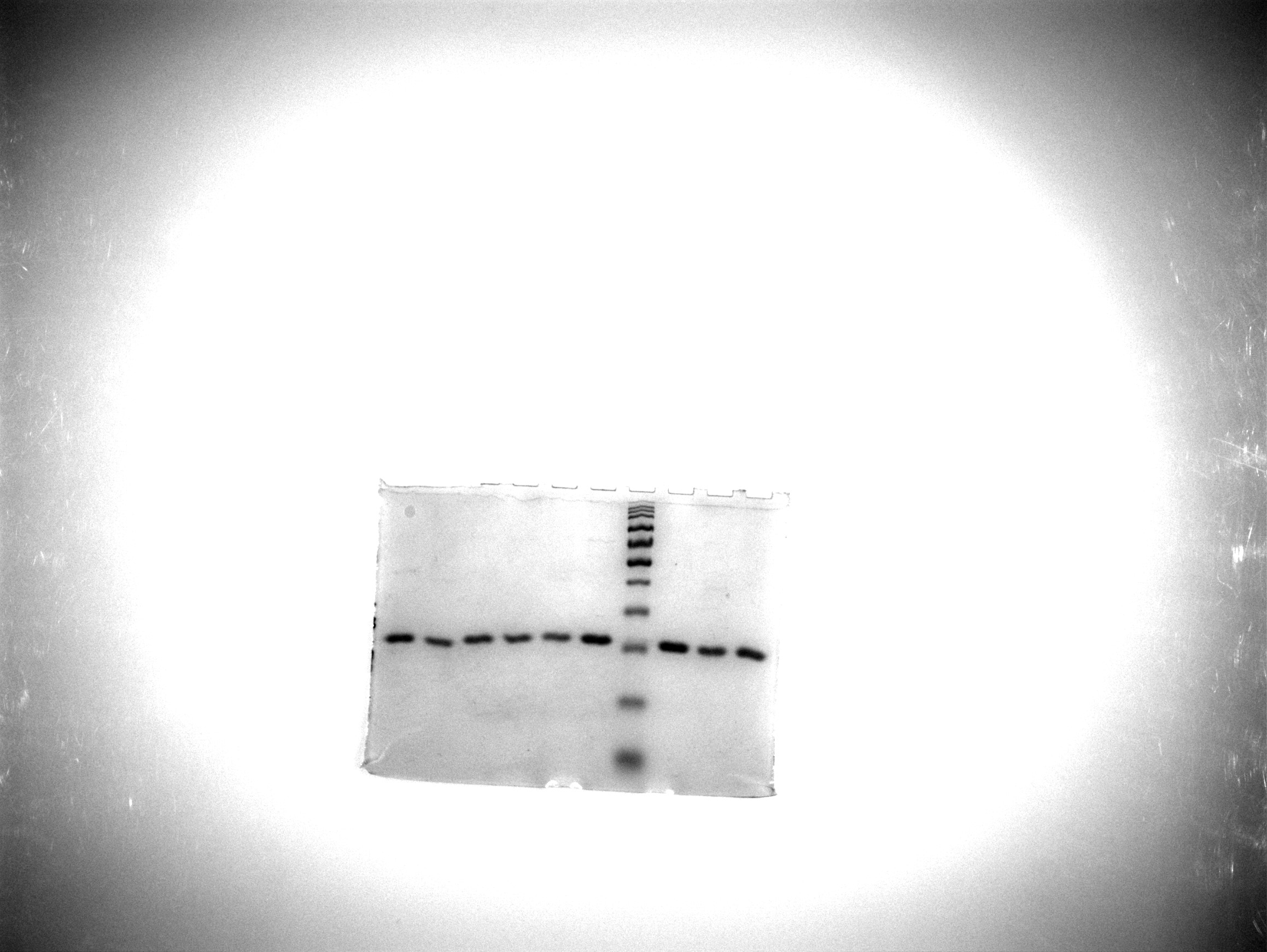

Supplement: Figure 1—figure supplement 1—source data 1. [file elife-80140-fig1-figsupp1-data1.zip › SDSgel.png]

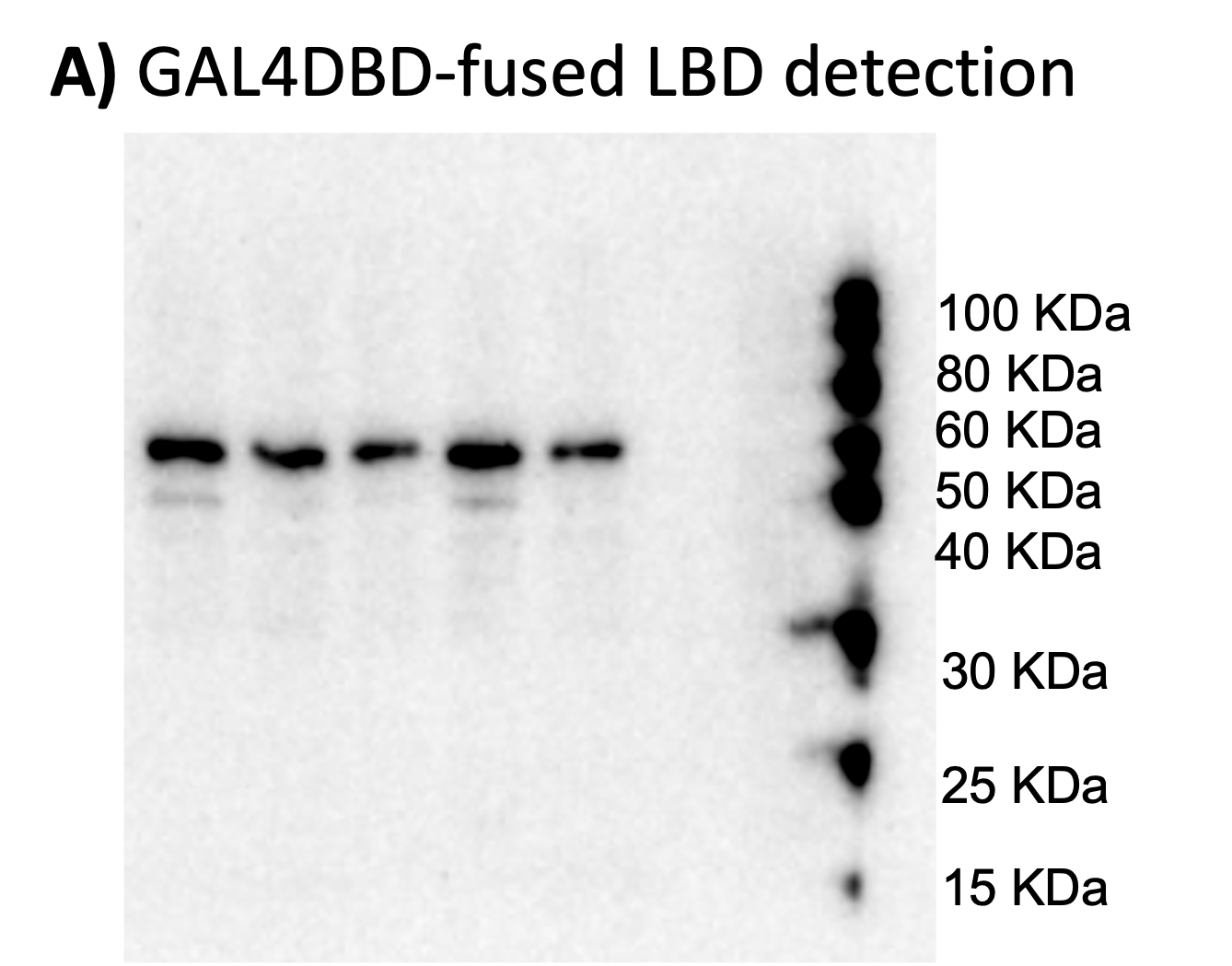

Supplement: Figure 2—figure supplement 2—source data 1. — An equal amount (40 μg) of total protein samples were electrophoresed on SDS-PAGE and transferred to a PVDF membrane. (A) Gal4-DBD fused protein was detected using ECI (Thermofisher scientific, USA) after incubation of mouse monoclonal anti-GAL4DBD antibody (sc510, Santacruz Biotechnology, Santa cruz, USA) and horse reddish peroxidase linked secondary antibody (sc-525409, Santacruz Biotechnology, Santa cruz, USA). Negative control was non-transfected Hela cells. (B) The same blot stripped off and then restained with rabbit raised anti-actin polyclonal antibody (A2066, Millipore Sigma, USA) and detected by Goat raised horse reddish peroxidase linked secondary antibody (ab97051, Abcam, USA). [file elife-80140-fig2-figsupp2-data1.zip › Blot1_labelled.png]

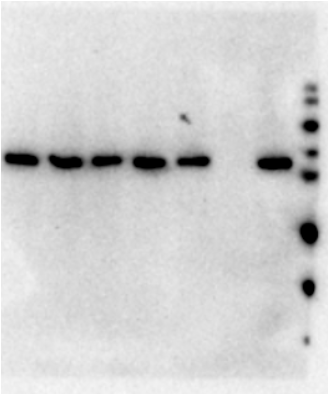

Supplement: Figure 2—figure supplement 2—source data 1. — An equal amount (40 μg) of total protein samples were electrophoresed on SDS-PAGE and transferred to a PVDF membrane. (A) Gal4-DBD fused protein was detected using ECI (Thermofisher scientific, USA) after incubation of mouse monoclonal anti-GAL4DBD antibody (sc510, Santacruz Biotechnology, Santa cruz, USA) and horse reddish peroxidase linked secondary antibody (sc-525409, Santacruz Biotechnology, Santa cruz, USA). Negative control was non-transfected Hela cells. (B) The same blot stripped off and then restained with rabbit raised anti-actin polyclonal antibody (A2066, Millipore Sigma, USA) and detected by Goat raised horse reddish peroxidase linked secondary antibody (ab97051, Abcam, USA). [file elife-80140-fig2-figsupp2-data1.zip › Blot2_actin.png]

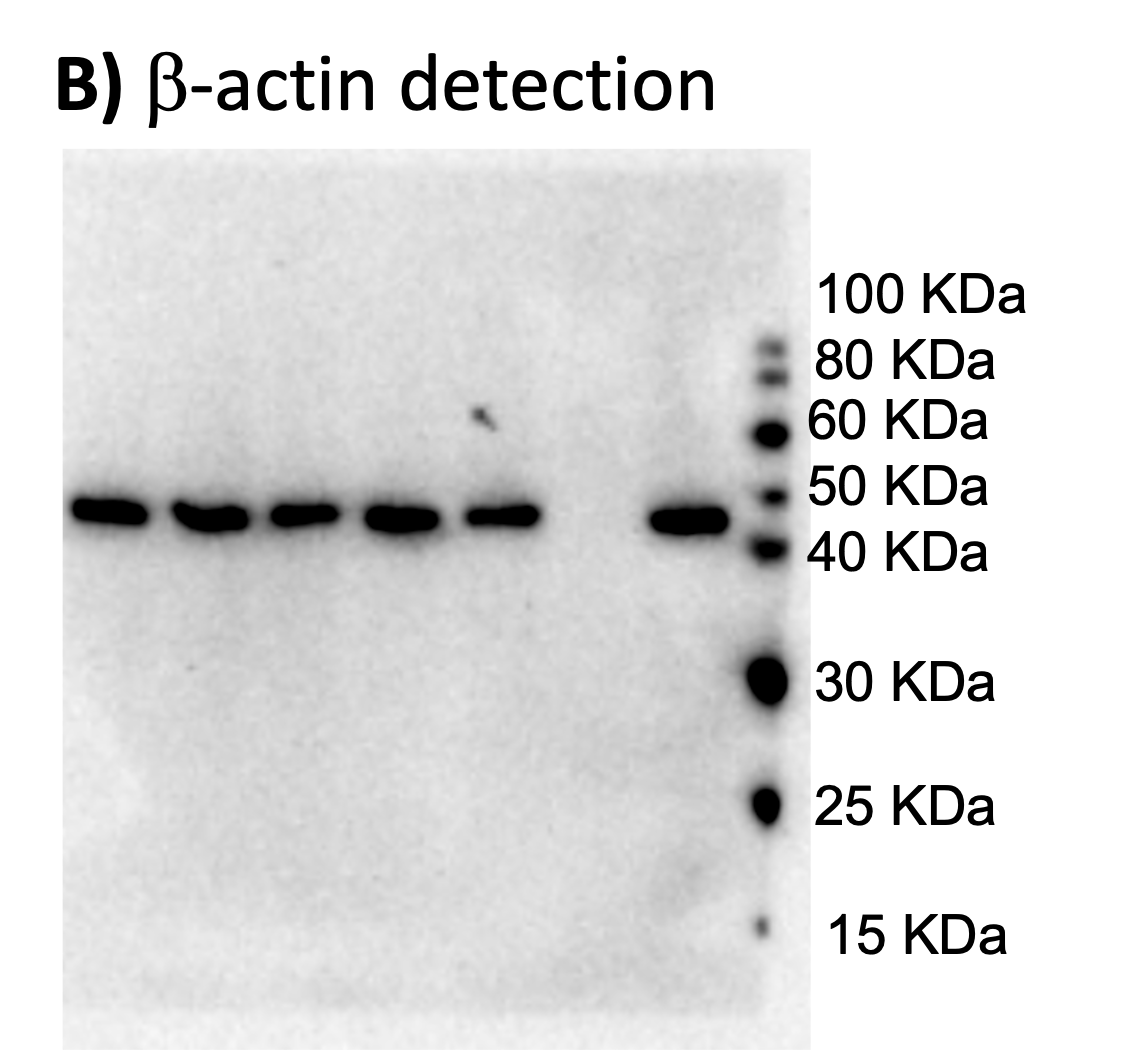

Supplement: Figure 2—figure supplement 2—source data 1. — An equal amount (40 μg) of total protein samples were electrophoresed on SDS-PAGE and transferred to a PVDF membrane. (A) Gal4-DBD fused protein was detected using ECI (Thermofisher scientific, USA) after incubation of mouse monoclonal anti-GAL4DBD antibody (sc510, Santacruz Biotechnology, Santa cruz, USA) and horse reddish peroxidase linked secondary antibody (sc-525409, Santacruz Biotechnology, Santa cruz, USA). Negative control was non-transfected Hela cells. (B) The same blot stripped off and then restained with rabbit raised anti-actin polyclonal antibody (A2066, Millipore Sigma, USA) and detected by Goat raised horse reddish peroxidase linked secondary antibody (ab97051, Abcam, USA). [file elife-80140-fig2-figsupp2-data1.zip › Blot2_labeled.png]

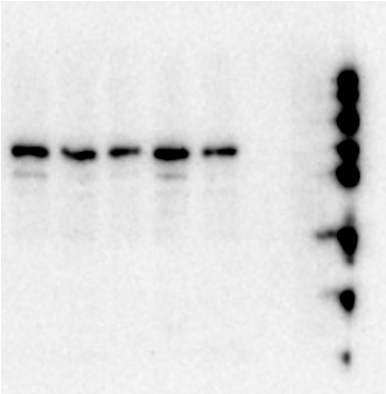

Supplement: Figure 2—figure supplement 2—source data 1. — An equal amount (40 μg) of total protein samples were electrophoresed on SDS-PAGE and transferred to a PVDF membrane. (A) Gal4-DBD fused protein was detected using ECI (Thermofisher scientific, USA) after incubation of mouse monoclonal anti-GAL4DBD antibody (sc510, Santacruz Biotechnology, Santa cruz, USA) and horse reddish peroxidase linked secondary antibody (sc-525409, Santacruz Biotechnology, Santa cruz, USA). Negative control was non-transfected Hela cells. (B) The same blot stripped off and then restained with rabbit raised anti-actin polyclonal antibody (A2066, Millipore Sigma, USA) and detected by Goat raised horse reddish peroxidase linked secondary antibody (ab97051, Abcam, USA). [file elife-80140-fig2-figsupp2-data1.zip › Blot1_gal4dbd.png]
