# Supplementary material for: Ligand-induced shifts in conformational ensembles that describe transcriptional activation
Source: eLife. 2022 Oct 12;11:e80140. doi: 10.7554/eLife.80140 (PMC9555869; doi:10.7554/eLife.80140)
Supplement: Supplementary file 1. [file elife-80140-supp1.docx]

| **Primer Name** | **Sequence** |
| --- | --- |
| **M75A_Forward** | 5’-CCTGGATGGGCCTGGCGGCCTTCGCCATGG-3’ |
| **M75A_Reverse** | 5’-CCATGGCGAAGGCCGCCAGGCCCATCCAGG-3’ |
| **M75I_Forward** | 5’-CCTGGATGGGCCTGATAGCCTTCGCCATG-3’ |
| **M75I_Reverse** | 5’-CATGGCGAAGGCTATCAGGCC CATCCAGG-3’ |
| **M75F_Forward** | 5’-CTGGATGGGCCTGTTCGCCTT CGCCATGG-3’ |
| **M75F_Reverse** | 5’ CCATGGCGAAGGCGAACAGGCCCATCCAG-3’ |
| **M75L_Forward** | 5'-CTGGATGGGCCTGTTGGCCTTCGCCAT-3’ |
| **M75L_Reverse** | 5’-ATGGCGAAGGCCAACAGGCCCATCCAG -3' |

**Table S1**: Oligonucleotide primers used for site-directed mutagenesis.
