# Supplementary material for: Ligand-induced shifts in conformational ensembles that describe transcriptional activation
Source: eLife. 2022 Oct 12;11:e80140. doi: 10.7554/eLife.80140 (PMC9555869; doi:10.7554/eLife.80140)
Supplement: Supplementary file 2. [file elife-80140-supp2.docx]

**Table S2**: Fractional populations for clusters

| Clusters | **Progesterone** | | **Estradiol** | | **DHT** | | **Aldosterone** | | **Cortisol** | |
| --- | --- | --- | --- | --- | --- | --- | --- | --- | --- | --- |
| M75A | APO | LIGANDED | APO | LIGANDED | APO | LIGANDED | APO | LIGANDED | APO | LIGANDED |
| 1 | 1.17% | 57.15% | 2.36% | 38.43% | 42.01% | 3.97% | 0.81% | 100.00% | 2.04% | 86.55% |
| 2 | 60.38% | 0.06% | 57.26% | 23.57% | 1.94% | 64.84% | 49.77% | 0.00% | 56.24% | 0.38% |
| 3 | 1.22% | 42.40% | 1.04% | 29.22% | 46.44% | 29.03% | 18.30% | 0.00% | 6.22% | 13.08% |
| 4 | 23.86% | 0.25% | 23.11% | 0.08% | 9.62% | 2.16% | 11.22% | 0.00% | 25.17% | 0.00% |
| 5 | 6.18% | 0.00% | 5.84% | 2.76% |  |  | 1.36% | 0.00% | 10.33% | 0.00% |
| 6 | 7.18% | 0.15% | 10.39% | 5.92% |  |  | 18.54% | 0.00% |  |  |
|  | **100%** | **100%** | **100%** | **100%** | **100%** | **100%** | **100%** | **100%** | **100%** | **100%** |
| M75F |  |  |  |  |  |  |  |  |  |  |
| 1 | 98.03% | 24.48% | 73.45% | 78.81% | 98.21% | 42.34% | 100.00% | 100.00% | 100.00% | 100.00% |
| 2 | 0.71% | 26.54% | 26.55% | 21.19% | 1.06% | 30.82% |  |  |  |  |
| 3 | 0.93% | 34.72% | 0.00% | 0.00% | 0.73% | 26.85% |  |  |  |  |
| 4 | 0.32% | 14.26% |  |  |  |  |  |  |  |  |
|  | **100%** | **100%** | **100%** | **100%** | **100%** | **100%** | **100%** | **100%** | **100%** | **100%** |
| M75L |  |  |  |  |  |  |  |  |  |  |
| 1 | 62.50% | 0.05% | 51.38% | 0.02% | 55.33% | 1.38% | 60.60% | 0.00% | 58.54% | 1.53% |
| 2 | 35.86% | 0.00% | 37.90% | 0.00% | 36.27% | 0.00% | 35.93% | 0.00% | 35.90% | 0.00% |
| 3 | 1.63% | 65.07% | 8.96% | 53.12% | 8.40% | 90.18% | 3.47% | 100.00% | 5.56% | 98.47% |
| 4 | 0.00% | 34.88% | 1.65% | 35.88% | 0.00% | 8.44% |  |  |  |  |
| 5 |  |  | 0.11% | 10.99% |  |  |  |  |  |  |
|  | **100%** | **100%** | **100%** | **100%** | **100%** | **100%** | **100%** | **100%** | **100%** | **100%** |
| AncSR2 |  |  |  |  |  |  |  |  |  |  |
| 1 | 93.07% | 2.80% | 80.71% | 61.41% | 99.12% | 0.14% | 92.37% | 0.01% | 90.16% | 0.01% |
| 2 | 6.93% | 97.20% | 19.29% | 38.59% | 0.88% | 53.05% | 7.63% | 99.99% | 9.84% | 93.89% |
| 3 |  |  |  |  | 0.00% | 44.17% |  |  | 0.00% | 6.10% |
| 4 |  |  |  |  | 0.00% | 0.97% |  |  |  |  |
| 5 |  |  |  |  | 0.00% | 1.68% |  |  |  |  |
|  | **100%** | **100%** | **100%** | **100%** | **100%** | **100%** | **100%** | **100%** | **100%** | **100%** |
